# Early Oral Feeding Compared With Traditional Postoperative Care in Patients Undergoing Emergency Abdominal Surgery for Perforated Duodenal Ulcer

**DOI:** 10.7759/cureus.12553

**Published:** 2021-01-07

**Authors:** Ayesha Masood, Sana Viqar, Naeem Zia, Muhammad usman Ghani

**Affiliations:** 1 General Surgery, California Institute of Behavioral Neurosciences & Psychology, Fairfield, USA; 2 Surgery, Benazir Bhutto Hospital, Rawalpindi, PAK; 3 Surgery, Rawalpindi Medical University, Rawalpindi, PAK

**Keywords:** perforated duodenal ulcer, eras protocols, randomized controlled trial, duodenal repair site leak, length of hospital stay, vas score, post-operative ileus

## Abstract

Introduction

Enhanced recovery after surgery (ERAS) protocols have been widely studied in elective abdominal surgeries with promising outcomes. However, the use of these protocols in emergency abdominal surgeries has not been widely investigated. This study aimed to evaluate ERAS application outcomes via early oral feeding compared to regular postoperative care in patients undergoing perforated duodenal ulcer repairs in emergency abdominal surgeries.

Materials and methods

We conducted a randomized controlled trial at the Surgical Unit 1 Benazir Bhutto Hospital from August 2018 to December 2019. A total of 42 patients presenting to the emergency department with peritonitis secondary to suspected perforated duodenal ulcer were included in the study. Patients were randomly assigned into two groups. Group A patients followed an ERAS protocol for early oral feeding, and Group B received regular postoperative care (i.e., delayed oral feeding). Our primary outcomes were the length of hospital stay, duodenal repair site leak, the severity of pain (via the visual analog scale), and postoperative ileus duration. Results were analyzed via IBM Statistical Product and Service Solutions (SPSS) Statistics for Windows, Version 20.0 (Armonk, NY: IBM Corp.). and chi-square and independent t-tests were applied.

Results

Patients who received early oral feeding (Group A) showed a shorter length of hospital stay, lower pain scores, and shorter postoperative ileus duration than patients in the traditional postoperative care group. Also, we noted no duodenal repair site leak in the early oral feeding group. The differences between the two groups were statistically significant (P<0.05).

Conclusions

Based on our results, ERAS protocols that promote early oral feeding can be applied in patients undergoing emergency abdominal surgery for perforated duodenal repair. Early oral feeding in emergency surgery patients can reduce the patient burden on hospitals. In addition, early oral feeding can promote better outcomes and reduced economic burden for patients. Keywords: Perforated duodenal ulcer, ERAS protocol, randomized controlled trial, duodenal repair site leak, length of hospital stay, VAS score, postoperative ileus.

## Introduction

A peptic ulcer is a common presentation found in medical outpatient departments. Nonsteroidal anti-inflammatory drugs and Helicobacter pylori infection are common causes of peptic ulcers, and most cases can be treated effectively with proton-pump inhibitors (PPIs) and eradication drugs for H.pylori [1]. Perforated duodenal ulcers are a severe complication of peptic ulcer disease with a mortality rate of up to 30% [2]. Surgical management of perforated duodenal ulcers is evolving, with laparoscopic techniques increasingly used, but open surgery is still a common strategy [3]. Enhanced recovery after surgery (ERAS) protocols have been used successfully in elective abdominal surgeries for decreased length of hospital stays and reduced postoperative morbidities and mortalities [4]. ERAS protocols have also been successfully applied in patients with colorectal malignancies undergoing resection and anastomosis with decreased rates of postoperative ileus and anastomosis site leak secondary to the beneficial effects of early oral feeding in terms of healing and reduced inflammatory response; this has led to decreased postoperative pain and ileus [5]. Recently, ERAS protocols have been applied in emergency colorectal surgeries, resulting in a shorter hospital stay and faster recovery from ileus [6]. A meta-analysis of randomized controlled trials on colorectal surgery reported the ERAS pathways' success in postoperative complications and mortality [7]. ERAS protocols have been widely studied in elective abdominal surgeries, but their impact on emergency abdominal surgeries is still being explored and may require modifications [8].

Currently, most of the patients in the emergency abdominal surgical setting follow traditional postoperative care protocols with late removal of nasogastric (NG) tubes and late start of oral feeding due to the fear of repair site leakage, which could cause more extended hospital stays and higher pain scores with delayed wound healing.

The goal of this study was to evaluate the effect of ERAS protocols in emergency abdominal surgeries in patients with perforated duodenal ulcers, specifically assessing the length of hospital stay, postoperative ileus, pain, and overcoming patient fears associated with perforation repair site leakage via early oral feeding.

## Materials and methods

We conducted a prospective, single-centre, randomized controlled double-blinded study to assess ERAS protocols' safety in patients with perforated duodenal ulcers undergoing emergency repair using Graham's patch repair or a modified Graham's patch repair. Ethical approval was provided by the Research Evaluation Unit of the College of Physicians and Surgeons Pakistan (REU NO: 33290). The study was also registered at www.clinicaltrials.gov (ClinicalTrials.gov Identifier: NCT04431037). Patients diagnosed with a perforated duodenal ulcer who presented to the emergency department of Benazir Bhutto Hospital Surgical Unit One from August 2018 to December 2019 were recruited after providing informed consent. The hospital staff was informed about the nature of the study and randomization. Patients were operated on in an emergency setting by third-year surgical residents or fourth-year residents.

According to their hospital registry number, patients were randomized into Group A and Group B using a simple random sampling technique via IBM Statistical Product and Service Solutions (SPSS) Statistics for Windows, Version 20.0 (Armonk, NY: IBM Corp.). A random number list was generated for 36 patients, randomly allocating them to Group A (17 patients) or Group B (19 patients). Every patient fulfilling the inclusion criteria was assigned a study ID number in chronological order to track the study in either Group A or Group B.

The surgical team was not informed before sampling, and patients were randomly sampled in the postoperative period. Patients in Group A received early oral feeding via a soft diet within 24 hours after surgery. Patients in Group B received regular postoperative care. They were allowed a soft diet after the passage of flatus or stools and the absence of air-fluid levels on abdominal radiographs. The surgical procedure elements, prophylactic antibiotics, and the surgeon performing the procedure was standardized to eliminate any potential confounding effect. Postprandial vomiting and abdominal pain measured by visual analog scale (VAS; scores >8) within 24h were treated with oral feeding suspension and antiemetic drugs and analgesics. If symptoms persisted for >24 hours, an NG tube was inserted. The surgical technique was standardized for both groups. Postoperative complications (e.g., abdominal pain), postoperative ileus (in terms of the number of days of return of bowel function), and prolonged hospital stay were recorded after the commencement of soft diet in both groups. Data were collected on standard forms prepared by researchers. Forms were completed by the attending trainees on call in the wards in the perioperative period.

The sample size was calculated. Inclusion criteria were all the patients older than 15 years with acute abdominal symptoms admitted in the emergency department suspected as a perforated duodenal ulcer. They operated within 24h of admission by the emergency department surgeon. Patients were excluded if they declined to join the study, had Peptic ulcers with both bleeding and perforation, had spontaneously sealed perforations, malignant ulcers, concurrent extra-abdominal surgery, preoperative need for endotracheal intubation, reoperation within one month, American Society of Anesthesiologists grade III/IV, or had an alternative perioperative diagnosis.

Preoperative care

Patients presented in the emergency department with suspected perforated duodenal ulcer underwent standard preoperative management with history taking, examination, and preoperative investigations. Patients started with intravenous fluids, antibiotics, and PPIs. An NG tube and Foley catheter were placed. Patients were resuscitated, and an exploratory laparotomy was conducted.

Perioperative surgical technique

**Figure 1:**
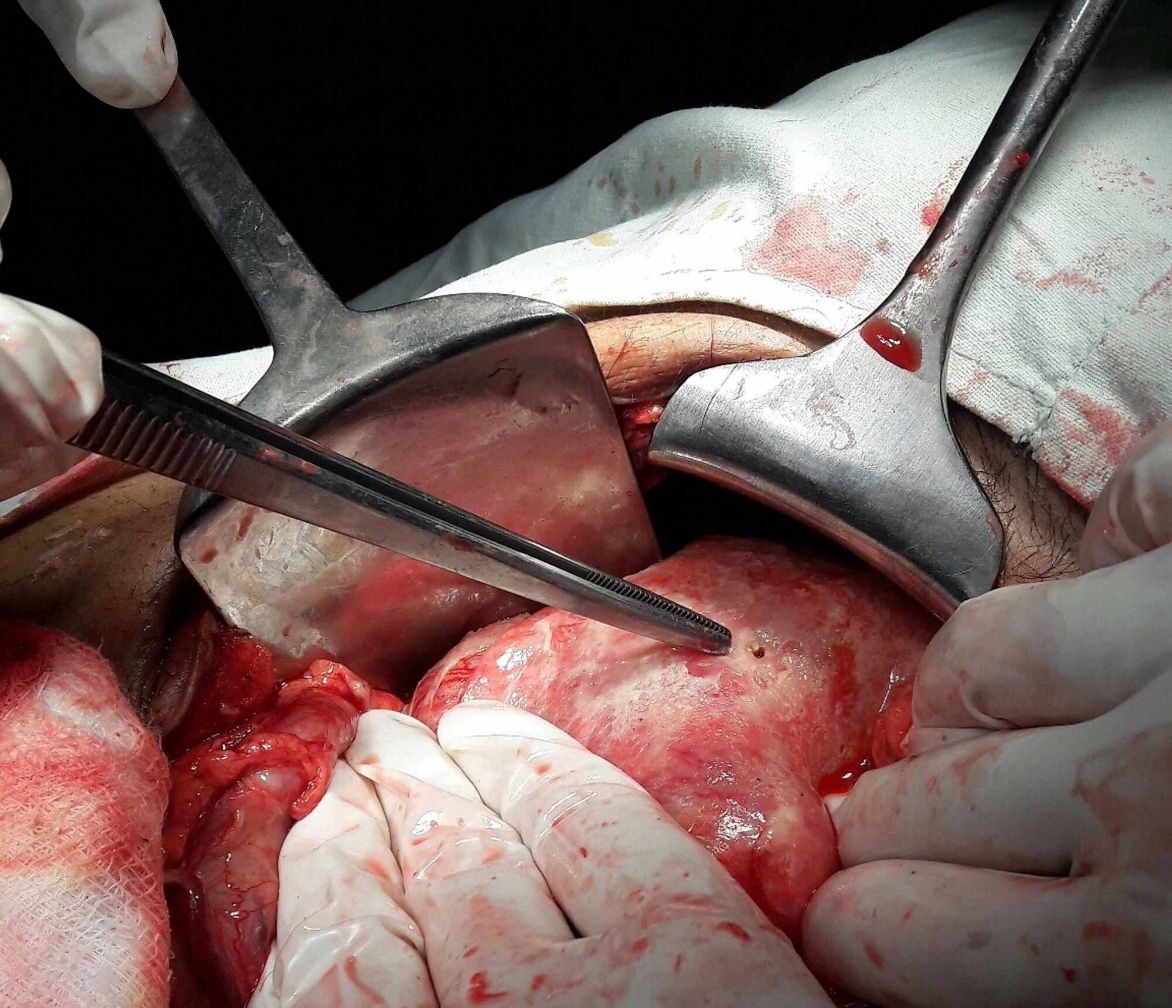
Perioperative image of perforated duodenal ulcer D1 area

Patients underwent either Graham's Patch Repair or Modified Graham's Patch Repair (Figure 1). Peritoneal lavage was performed with normal saline to remove collections and debris, and the abdomen was then closed.

Postoperative technique

Group A patients (the ERAS Group) were admitted in the high dependency unit (HDU) postoperatively. The NG tube and Foley catheter were removed within 12 hours, and patients were allowed oral sips on day one with a gradual shift to liquid diet after 12 hours; semisolid food was started after 24 hours. Patients were given intravenous (IV) antibiotics, painkillers, and IV PPIs and shifted to oral pain killers on the second postoperative day (POD; Table 1).

**Table 1:** Postoperative care protocol Abbreviations: HDU, high dependency unit; NG, nasogastric.

	Group A	Group B
Early Postoperative Admission	HDU	HDU
Removal of NG tube	Within 12hours	After 48 hours
Removal of Foley catheter	Within 12 hours	After 24 hours
Time to allow oral sips	After 12 hours	After 48 hours
Time to allow liquid diet	After 18 hours	After 72 hours
Time to allow solid/semisolid diet	After 24 hours	After 72 hours
Mobilization of the patient	After 12 hours	After 24 hours
Shift to oral painkillers	On second postoperative day	On third or fourth postoperative day

Group B patients (the traditional postoperative care group) were admitted to the HDU. The Foley catheter and NG tube remained for 48 hours following surgery, and patients remained nil per os (NPO; i.e., nothing by mouth) for three days and started with oral sips after 72 hours. They were given maintenance fluids and adjusted according to fluid losses from the NG tube. Patients were given IV antibiotics and PPIs during the hospital stay with IV pain killers and antiemetics until the second POD. Patients were discharged on the full resumption of diet, with regular bowel activity and normal total leukocyte count, in vitally stable condition and called for follow-up after seven to 10 days.

Endpoints

The study's primary endpoints were hospital stay length, pain score on the VAS, number of days of return of bowel function, and ulcer repair site leak. Secondary endpoints were the need for nasogastric tube reinsertion, readmission rate after discharge, and vomiting. Patients were monitored for readmission in-person or via phone (if they went to a different hospital).

Statistical analysis

All data were entered and analyzed using IBM SPSS Statistics for Windows, Version 20.0 (Armonk, NY: IBM Corp.). The categorical values like gender and types of surgery were expressed as frequency or percentages. The quantitative variables like age, postoperative pain, length of hospital stay, and days of return of bowel function were expressed as mean or standard deviation. The student's t-test was applied to compare hospital stay, days of return of bowel function, and pain score between them. Effect modifiers like age and gender, and type of surgery were controlled by stratification, and post-stratification students' t-test was applied. A p-value of<0.05 was considered statistically significant.

## Results

**Figure 2:**
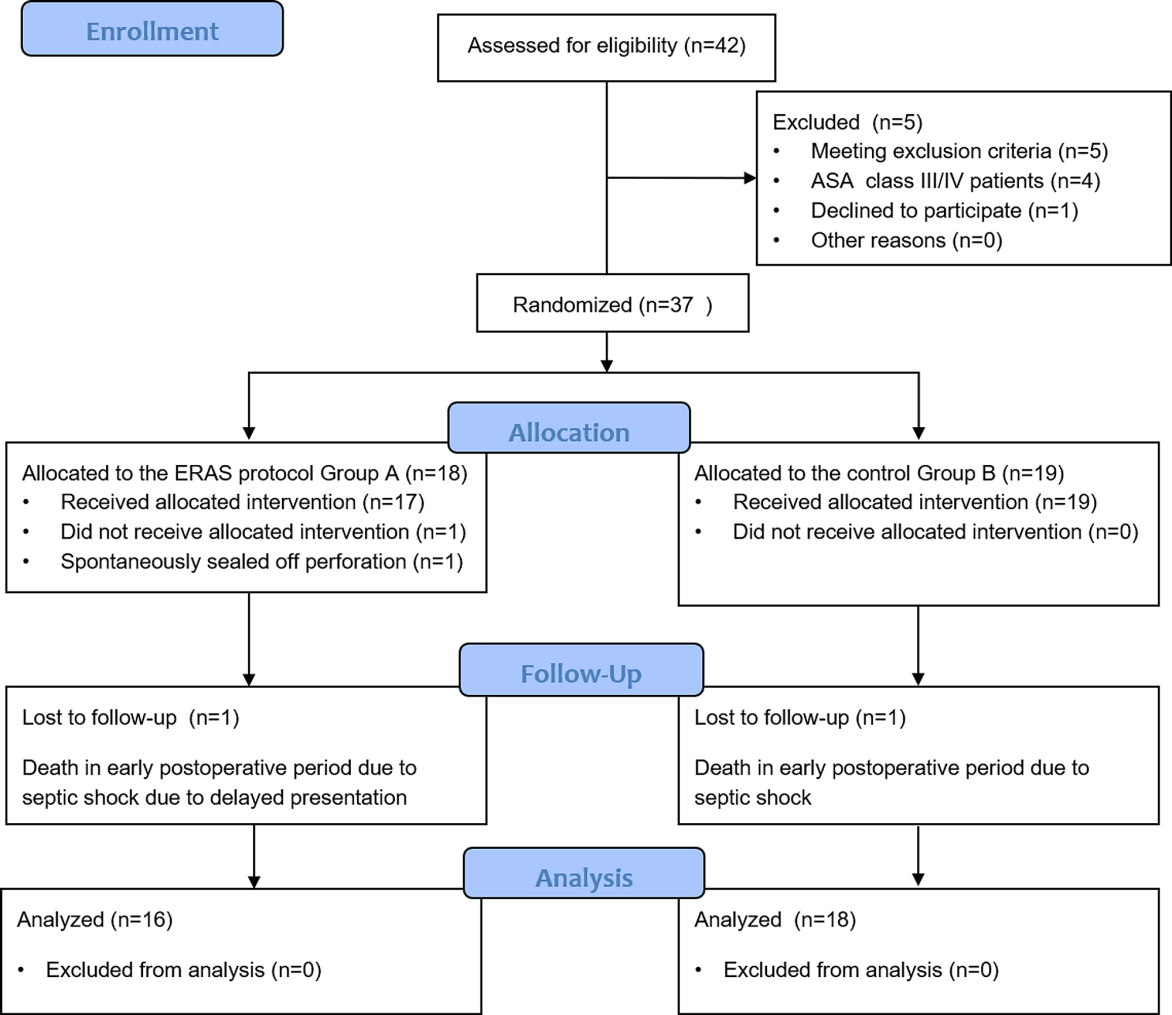
CONSORT Flow diagram ERAS- Enhanced recovery after surgery; CONSORT- Consolidated Standards and Reporting of Trials

Figure 2 presents the enrollment process details as a Consolidated Standards and Reporting of Trials (CONSORT) Flow Diagram.

A total of 36 cases fulfilling the selection criteria were enrolled to compare the outcomes of early oral feeding with traditional postoperative care after emergency abdominal surgeries in terms of length of hospital stay, postoperative pain, time to return of bowel function, and repair site leak (Table 2).

**Table 2:** Distribution of patients according to outcome variables

Outcomes		Group A	Group B
Length of stay	≤4 days	12	0
	>4 days	4	18
Postoperative pain	Mild pain	16	0
	Moderate to severe pain	0	18
Return of bowel function	Within 24 hours	15	3
	After 24 hours	1	15
Repair site leak		None	None

Of the 36 total patients in the study, 17 received early oral feeding (16 male patients, 94.1%; one female patient, 5.9%), and 19 received traditional postoperative care (16 male patients, 84.2%; three female patients, 15.8%; Table 3).

**Table 3:** Demographic profiles of study participants (a) Fischer’s Exact test; (b) Independent t-test; (c) Chi-square test Abbreviation: SD, standard deviation.

		Group A (n=17)	Traditional Postoperative Group B (n=19)	p-value
Gender	Male	16 (94.1%)	16 (84.2%)	0.605 (a)
	Female	1 (5.9%)	3 (15.8%)	
Age	Mean (SD)	52.59 (20.49)	49.68 (17.51)	0.650(b); 0.813( c)
	<40 years	6 (35.3%)	6 (31.6%)	
	≥40 years	11 (64.7%)	13 (68.4%)	

After excluding the two patients who died due to septic shock and ERAS protocols could not be applied, the data from 34 patients were analyzed.

**Figure 3:**
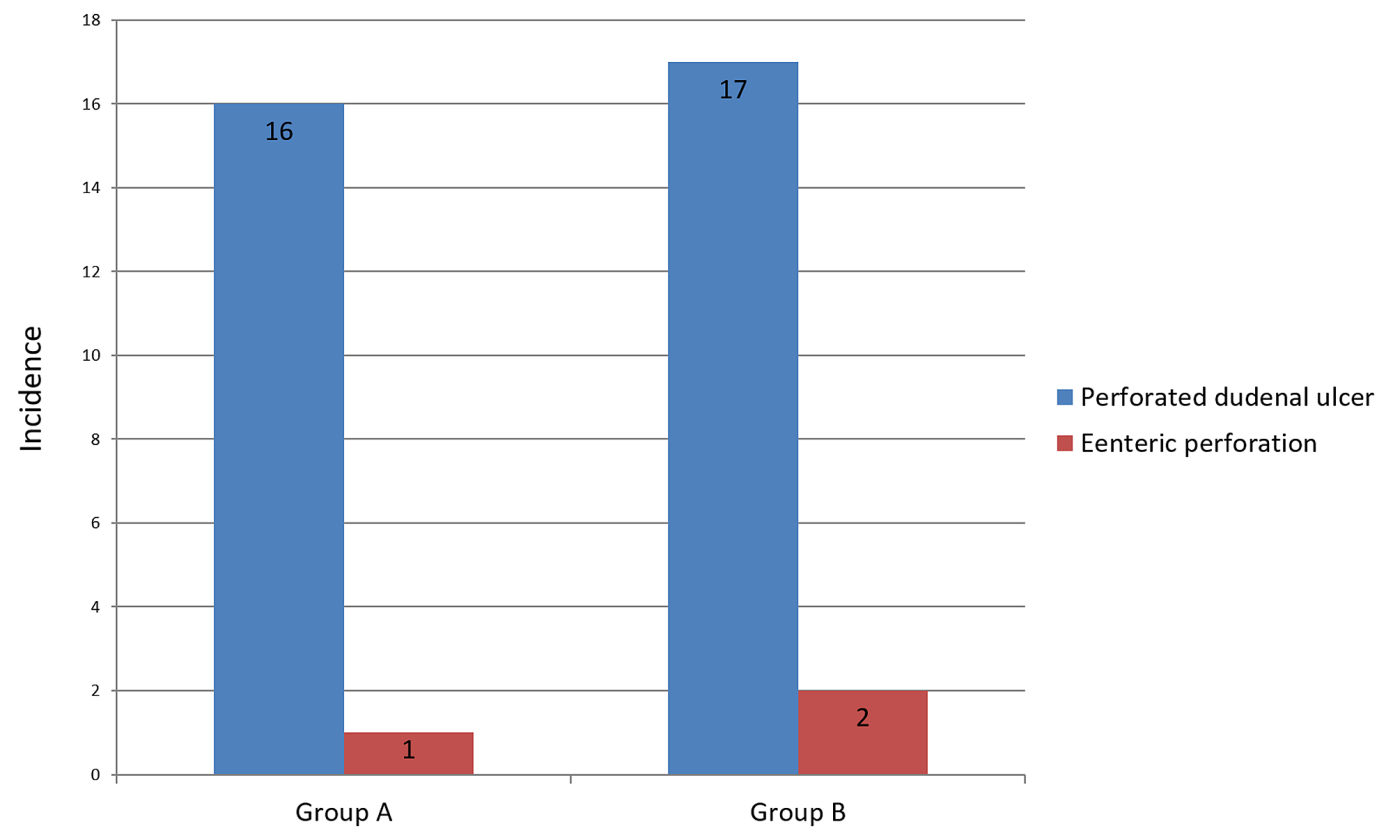
Diagnosis at admission for early oral feeding group and delayed oral feeding group patients

Fischer's exact test was applied for gender distribution (p=0.605). Patients were grouped into two categories according to age to see the prevalence of peptic ulcer by age. The early oral feeding group contained six patients (35.3%) younger than 40 years and 11 patients (64.7%) age 40 or older (mean age, 52.5 ± 20.4 years). The traditional postoperative care group had six patients (31.6%) younger than 40 years and 13 patients (68.4%) age 40 or older (mean age, 49.6 ± 17.5). Independent t-test (p=0.650) and Chi-squared tests (p=0.813) were applied. Thirty-three patients (91.7%) had suspected perforated duodenal ulcer on admission, and three (8.3%) had enteric perforation postoperatively (Figure 3).

A total of 15 patients had <500mL of perioperative pus. Eight of those (47.1%) were in the early oral feeding group, and seven (38.9%) were in the traditional postoperative care group. A total of 20 patients had >500 mL pus: nine (52.9%) in the early oral feeding group and 11 (61.1%) in the traditional care group (p=0.625). One patient in the traditional care group presented with a bleeding peptic ulcer and the amount of pus could not be assessed due to the bleeding. This patient was excluded from the analysis.

**Figure 4:**
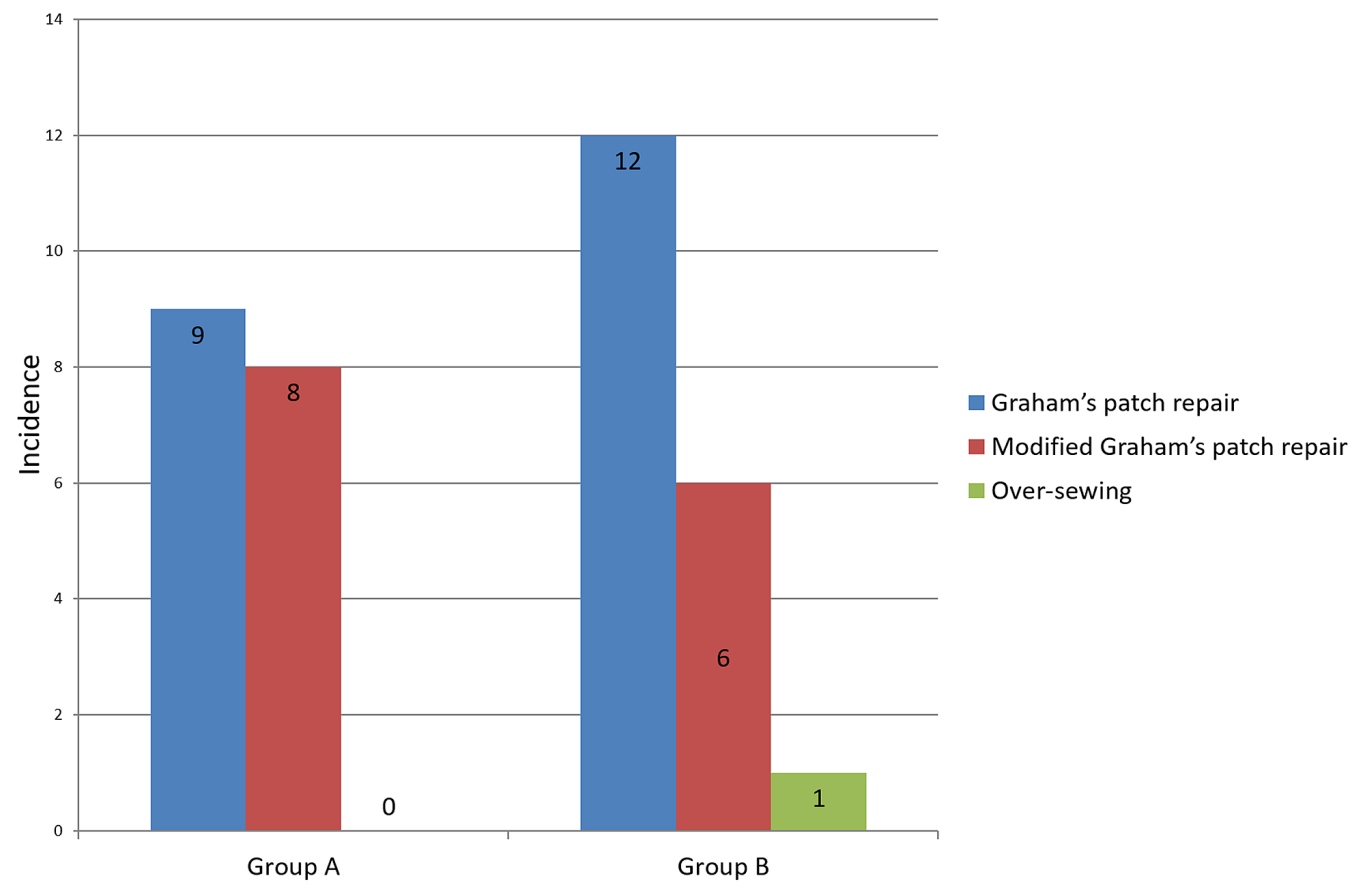
Repair technique incidence for early oral feeding and delayed oral feeding groups

Thirteen total patients had perioperative perforation size <0.5×0.5cm; five were in the early oral feeding group, eight were in the traditional care group. Twenty-three patients had perioperative perforation size of >0.5×0.5cm, with 12 in the early oral feeding group and 11 in the traditional care group (p=0.429). Modified Graham's Patch repair was done in 14 patients, and simple Graham's Patch repair was done in 21 patients. Over sewing of the posterior duodenal wall was performed in one patient with a bleeding ulcer (Figure 4, Table 4).

**Table 4:** Peroperative findings (a) Amount of pus could not be assessed due to bleeding in one patient in Group B (delayed oral feeding); this patient was excluded from the analysis.

Findings		Group A (n=17)	Group B (n=19)	p-value
Size of perforation	<0.5cm	5(29.4%)	8(42.1%)	0.429
	>0.5cm	12(70.6%)	11(57.9%)	
Amount of pus (a)	<500 mL	8(47.1%)	7(38.9%)	0.625
	>500 mL	9(52.9%)	11(61.1%)	

Early oral feeding was started after 12 hours in 16 patients, and traditional postoperative care was given to 18 patients. In the early oral feeding group, the mean length of hospital stay was four days (range, two to 10 days). The mean length of hospital stay was six days (range, five to 10 days; p=.000). The mean time to return of bowel function in the early oral feeding group was 24 hours (range, 12 to 48 hours), and the traditional care group had a mean time to return of bowel function of 48 hours (range, 48 to 96 hours; p=.000). In the early oral feeding group, the mean pain score was three (range, two to seven) than the traditional care group's mean pain score of eight (range, four to nine; p=.000). We applied the Mann-Whitney U test for analysis (Table 5).

**Table 5:** Comparison of outcome variables

Outcomes	Group A (n=16)	Group B (n=18)	p-value
Return of bowel function (hours)	24 (12-48)	48 (24-96)	0.000
Length of hospital stay (days)	4 (2-10)	6 (5-10)	0.000
Postoperative pain score (visual analogue scale)	3 (2-7)	8 (4-9)	0.000

**Figure 5:**
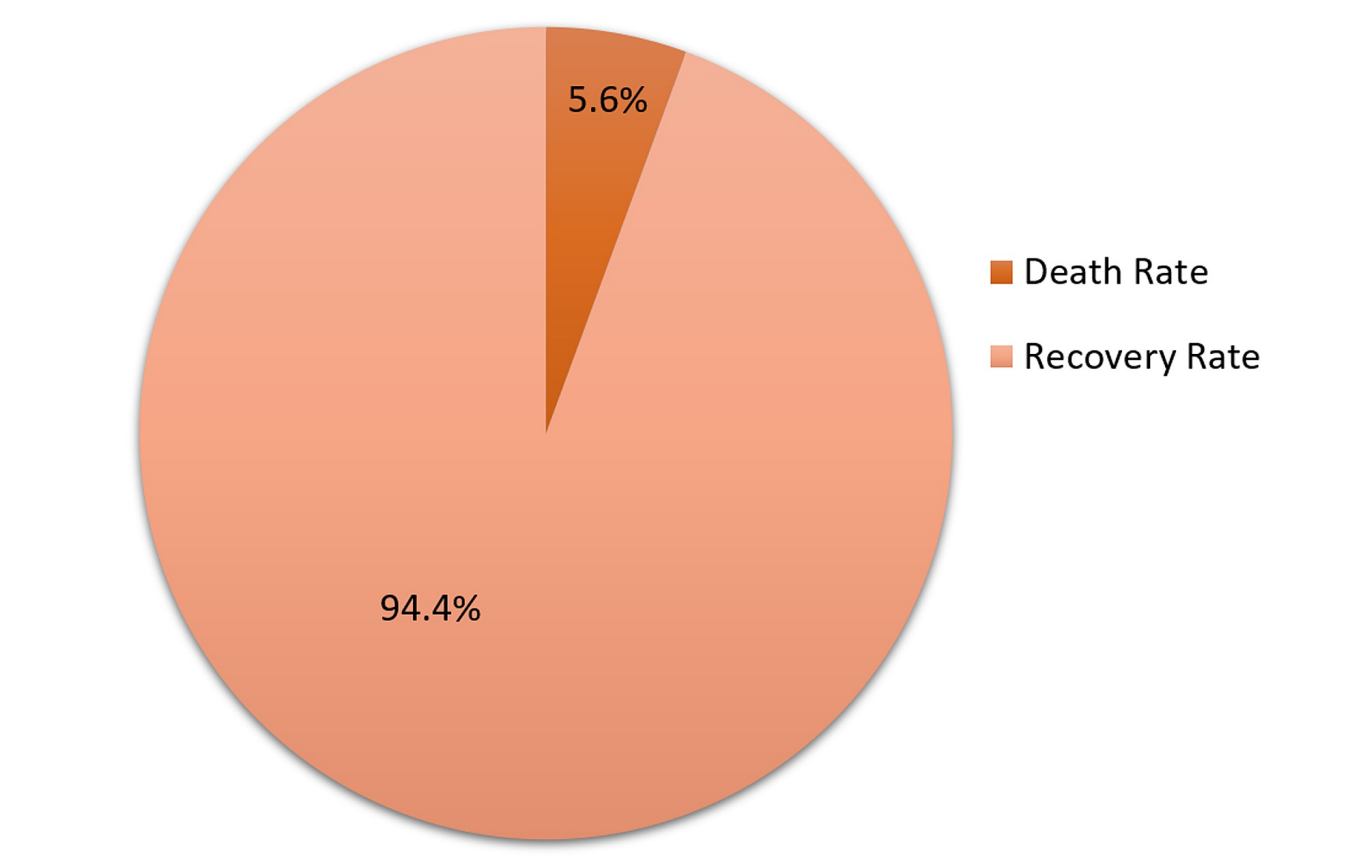
Mortality rate vs recovery rate

Two patients were admitted to the intensive care unit (ICU) on ventilatory support and could not be assessed for these parameters because of their critical condition and early postoperative mortality. Regarding complications, we found no repair site leak in either group (Figure 5).

**Figure 6:**
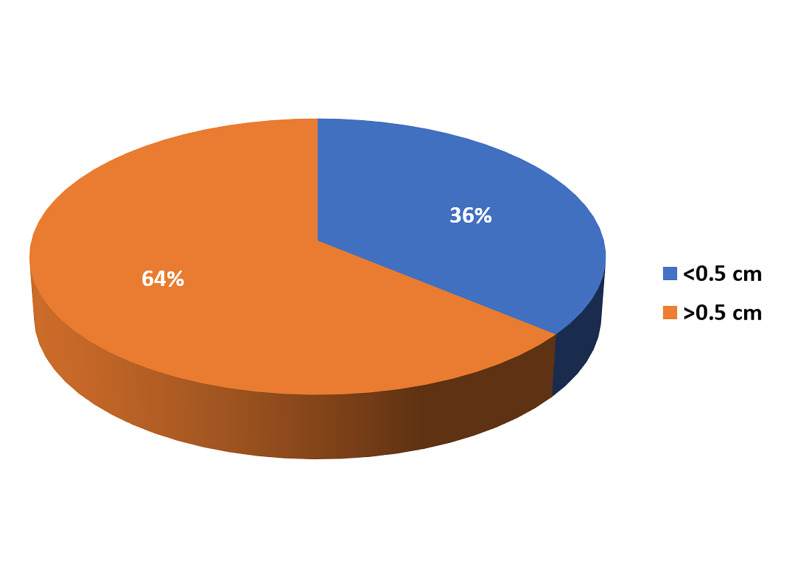
Size of perforation

One patient in the traditional postoperative care group developed diarrhoea (2.8%). This patient was managed conservatively and discharged in stable condition. A burst abdomen was seen in one patient (2.8%) in the early postoperative care group confirmed by ultrasound as not associated with the intraabdominal collection. For this patient, an abdominal wash and reclosure were performed, and the patient was discharged in stable condition on POD 10. Chest infection was noted in two patients (5.6%) in the traditional postoperative care group with atelectasis on chest x-ray, fever with crepitus in the lung fields, and productive cough. These patients were managed with chest physiotherapy and nebulizations, along with appropriate antibiotics. (Figure 6).

**Figure 7:**
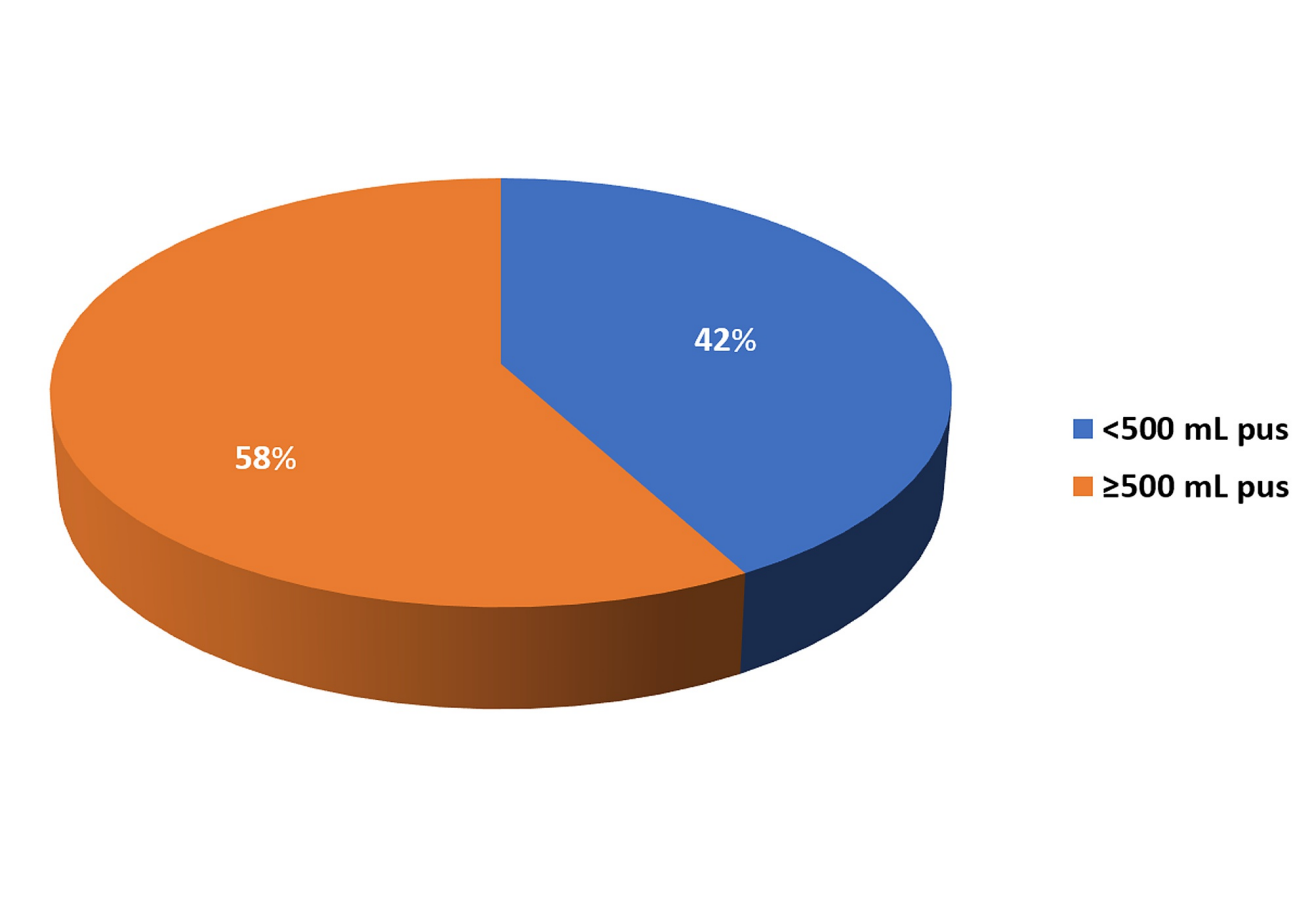
Amount of pus

Of the 16 patients in Group A, eight had <500 mL pus and mild postoperative pain (i.e., <5 on VAS), and eight patients had >500 mL pus with moderate to severe levels of pain (>5 on VAS). Of the 19 patients in Group B, seven had >500 mL pus with a pain moderate to severe pain (>5 on VAS), and 12 patients had <500 mL pus and reported no pain. Therefore, the amount of pus was not associated with pain intensity in this study (Figure 7).

**Figure 8:**
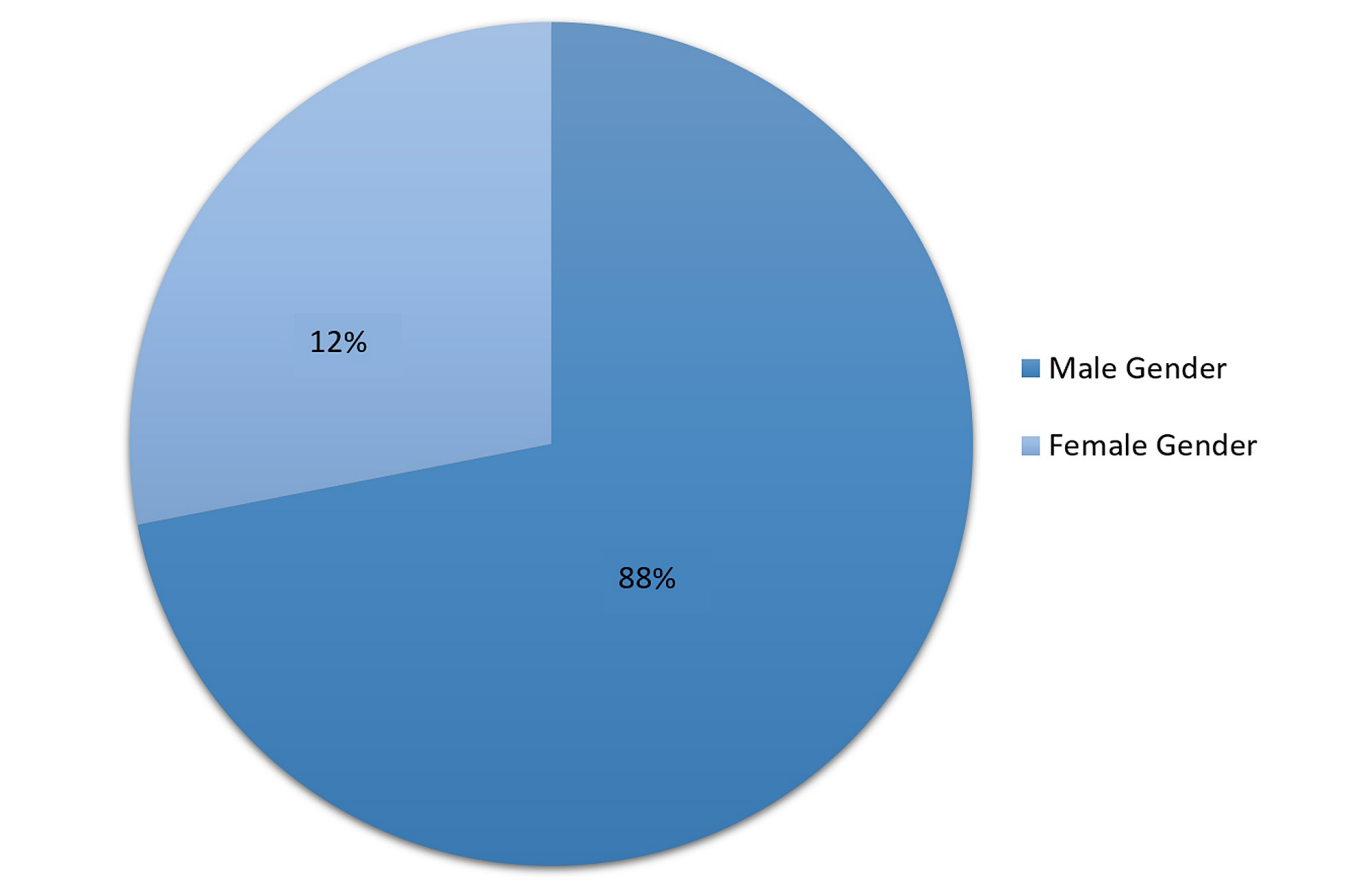
Gender distribution

Most patients were age 40 years or older (n=24; 66.7%), and perforated duodenal ulcer was more common in the older age group than the younger age group (Figure 8).

Younger patients (those younger than 40 years, n=12) had a mean hospital stay of 5.25 ± 2.2 days, and those aged 40 and older had a mean hospital stay of 5.14 ± 1.67 days. Therefore, the length of hospital stay was not associated with age in this study.

## Discussion

Traditional postoperative care has been practised for many years in elective and emergency abdominal surgeries with protocols that allow oral feeding after three to five days following perforated duodenal ulcer repair or gut anastomosis. This protocol allows the repair site to heal and normal gut peristalsis to resume, so repair site leak chances are minimized [9]. Part of the reason why this approach has been so broadly adopted is that repair site leak is a surgeon's nightmare. Perforated duodenal ulcer repair leak presents a significant challenge for surgeons to manage and can be disastrous for the patient [10]. Given the evolving knowledge of pathophysiology and constant research on ERAS pathways, there is a major shift towards breaking NPO as early as the patient becomes conscious in the recovery room after anaesthesia. In almost all elective surgeries except esophageal anastomosis, starting early oral feeding has had promising results regarding patient benefits and lower health system costs [11]. There have also been successful outcomes in emergency surgeries in terms of repair site leak [12]. This study evaluated the effects of early oral feeding in patients with perforated duodenal ulcers to see ERAS pathways' benefits and hazards. The anticipation that further research in this field would be conducted.

In our study, the repair technique was done per the operating surgeon's preference, and there were no differences noted in repair site leakage, consistent with previous studies [13]. However, two patients in our study presented with septic shock as referred cases received surgery and were admitted to the ICU postoperatively on ventilator support. They died on the second and third days following surgery. Late presenting perforated duodenal ulcer with septic shock has a high mortality, which underlines the importance of early detection of the symptoms and initiating preventive measures against the causative agents.

We found that postoperative ileus was significantly lower in the early oral feeding group than the traditional postoperative care group. Because inclusion and exclusion criteria were identical for both groups, the early return of bowel function can be attributed to the ERAS protocol. The use of the NG tube for a prolonged period leads to prolonged ileus [14].

Postoperative pain scores were lower in the ERAS early feeding group than the traditional postoperative care group. Another study reported that ERAS patients used fewer opioid analgesics resulting in early gut motility and reduced length of hospital stay with better food tolerance [15]. Our study results are in favour of the use of ERAS protocols to reduce postoperative pain.

Most of the study participants were older than 40 years, showing that disease is more prevalent among older age groups [16]. Perforated duodenal ulcers were more prevalent among men than women, consistent with previous studies [17].

ERAS pathways can be used in emergency surgery cases where there is a risk of repair site leak in perforated duodenal ulcer. Hospital stay duration was significantly less in the early oral feeding group than the traditional postoperative care group, similar to previous studies [13,18]. Early oral feeding after perforated duodenal ulcer repair did not result in the repair site leak in any patients. A previous study reported that primary small bowel anastomosis in emergency cases also showed encouraging results if patients are properly selected and operated upon by experienced surgeons [9].

Limitations

Our study had several significant limitations. The study sample size was relatively small. We could not adequately assess the percentage of patients who experienced postoperative nausea and vomiting with each successive meal. Also, the study's nature exploring emergencies did not permit us to follow proper preoperative ERAS protocols. Patients with American Society of Anesthesiologists Grade III/IV status were not included in the study. Future studies should account for this to better determine the efficacy of ERAS in emergency abdominal surgeries.

## Conclusions

Prolonged use of NG tubes and NPO status for longer than 24 hours is associated with a prolonged hospital stay, postoperative ileus, and increased pain scores. We found no leak from the repair sites for patients who started early oral feeding. ERAS that promote early oral feeding can be safely applied in selected patients undergoing emergency surgeries for perforated duodenal ulcers. Early oral feeding in emergency surgery patients can reduce the patient burden on hospitals and promote better outcomes and reduced economic burden.
